# Toxic epidermal necrolysis induced by exposure to S,S-dimethyl cyanoimidodithiocarbonate: case report and nursing experience

**DOI:** 10.3389/fmed.2026.1750451

**Published:** 2026-05-28

**Authors:** Xue-Qing Liu, Xiao-Lan Dong, Yue-Tong Qian, Chang Shu

**Affiliations:** Department of Dermatology, Peking Union Medical College Hospital, Chinese Academy of Medical Sciences & Peking Union Medical College, Beijing, China

**Keywords:** care, nursing, nursing care, S,S-dimethyl cyanoimidodithiocarbonate, toxic epidermal necrolysis

## Abstract

This study summarizes the personalized diagnosis, treatment, and nursing process of a patient with toxic epidermal necrolysis (TEN) caused by exposure to S,S-dimethyl cyanoimidodithiocarbonate (DCT). Key treatment regimens include systemic glucocorticoids, cyclosporine, intravenous immunoglobulin, and single-dose infliximab, combined with nursing care for impaired skin and mucosal integrity, multidisciplinary pain management, and supportive therapy. The patient had approximately 60% body surface area (BSA) involvement upon admission. After 20 days of intensive treatment and personalized nursing, the patient’s skin lesions had nearly healed, and the condition improved sufficiently to allow discharge.

## 1 Introduction

Toxic epidermal necrolysis (TEN) is a severe skin and mucosal reaction characterized by blistering and widespread epidermal detachment, often accompanied by systemic symptoms, including multiorgan failure syndrome (1). A U.S. study indicated (2) that although TEN has a low incidence, its mortality rate is high (14.8%). The majority of TEN cases are drug-induced (3), commonly caused by anticonvulsants, antidepressants, non-steroidal anti-inflammatory drugs, anti-infective agents, and, more recently, targeted drugs. TEN caused by chemical agents is extremely rare. DCT is a chemical compound synthesized from calcium cyanamide, carbon disulfide, and dimethyl sulfate, all of which are toxic. Calcium cyanamide may cause dermatitis, urticaria, and ulcers. Carbon disulfide and dimethyl sulfate can be absorbed through the respiratory tract and skin; carbon disulfide can cause poisoning, while dimethyl sulfate hydrolyzes into sulfuric acid, leading to corrosive tissue damage. Severe cases may present with diffuse edematous erythema, flaccid blisters, bullae, a positive Nikolsky’s sign, and epidermal detachment resembling burns. Gentle rubbing can cause large areas of epidermal stripping, exposing red erosive surfaces, often accompanied by fever (4, 5).

In August 2023, our department admitted a patient with TEN caused by exposure to DCT. The epidermal detachment involved more than 60% of the body surface area. The patient was at high risk of infection and also obese, and the unique etiology made treatment and nursing particularly challenging. Drawing on our prior experience with multiple TEN patients, we implemented 20 days of intensive treatment and targeted nursing care, resulting in nearly complete healing of skin lesions and improved condition at discharge. The nursing experience is summarized below:

## 2 Clinical data

The patient developed local erythema and pruritus after exposure to “DCT powder.” The following day, several millet-sized blisters with clear fluid and thin walls appeared, which ruptured easily (Table 1). He first sought treatment at a local hospital. During this period, hormones, antibiotics, and traditional Chinese medicine treatments were administered. However, after 20 days of therapy, his skin lesions were not effectively controlled and continued to progress, accompanied by a high fever (Tmax: 39.3 °C). He then presented to our hospital’s emergency department and was admitted to the dermatology ward (Table 2). On admission, the patient was diagnosed with TEN, abnormal liver function, fatty liver, splenomegaly, and obesity. The patient’s disease course is as follows:

**Table 1:** Basic patient information.

Item	Specific information
Age	20 years old
Sex	Male
Height	175 cm
Weight	103 kg
Medical history	Denies a history of chronic medical such as high blood pressure, coronary heart disease, and diabetes mellitus. Denies a history of infectious diseases, including hepatitis, tuberculosis, typhoid fever, and malaria. Denies a history of major surgeries, trauma, or blood transfusion. Denies a history of drug allergies. Denies a recent history of vaccination.
Family history	The patient’s father has a history of metal-induced contact allergy.
Psychosocial background	Following the onset of the illness, the patient experienced significant anxiety due to pain and alterations in appearance. His parents serve as the primary caregivers, and family support is strong.
Occupational/social stressors	The patient works as a freight truck driver. He reported exposure to DCT powder without the use of occupational protective measures.
Relevant genetic predispositions	No genetic testing (e.g., HLA genotyping) was performed for this patient.

**Figure 1:**
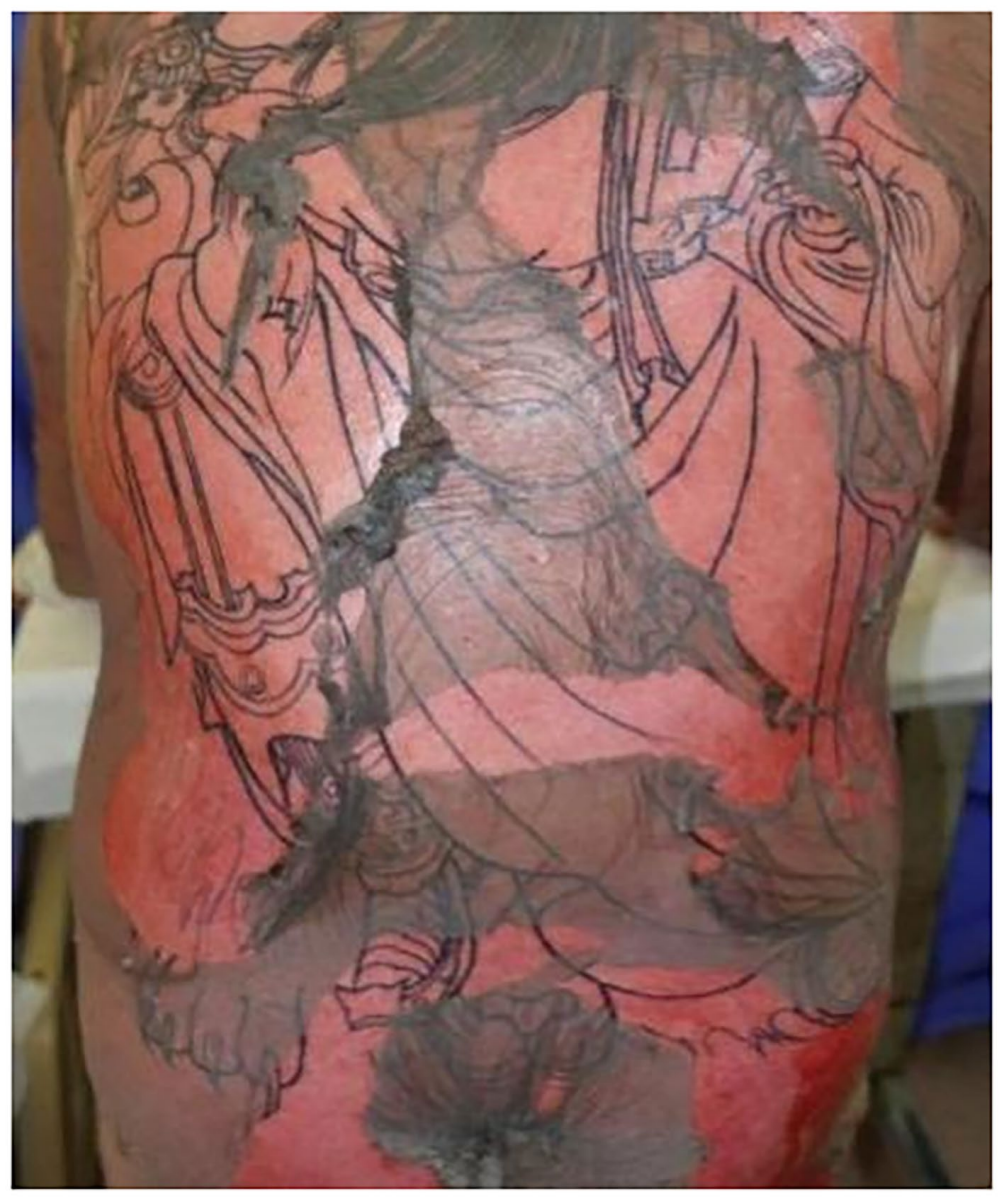
Clinical appearance of toxic epidermal necrolysis. Generalized erythema and epidermal detachment were apparent on the trunk.

**Figure 2:**
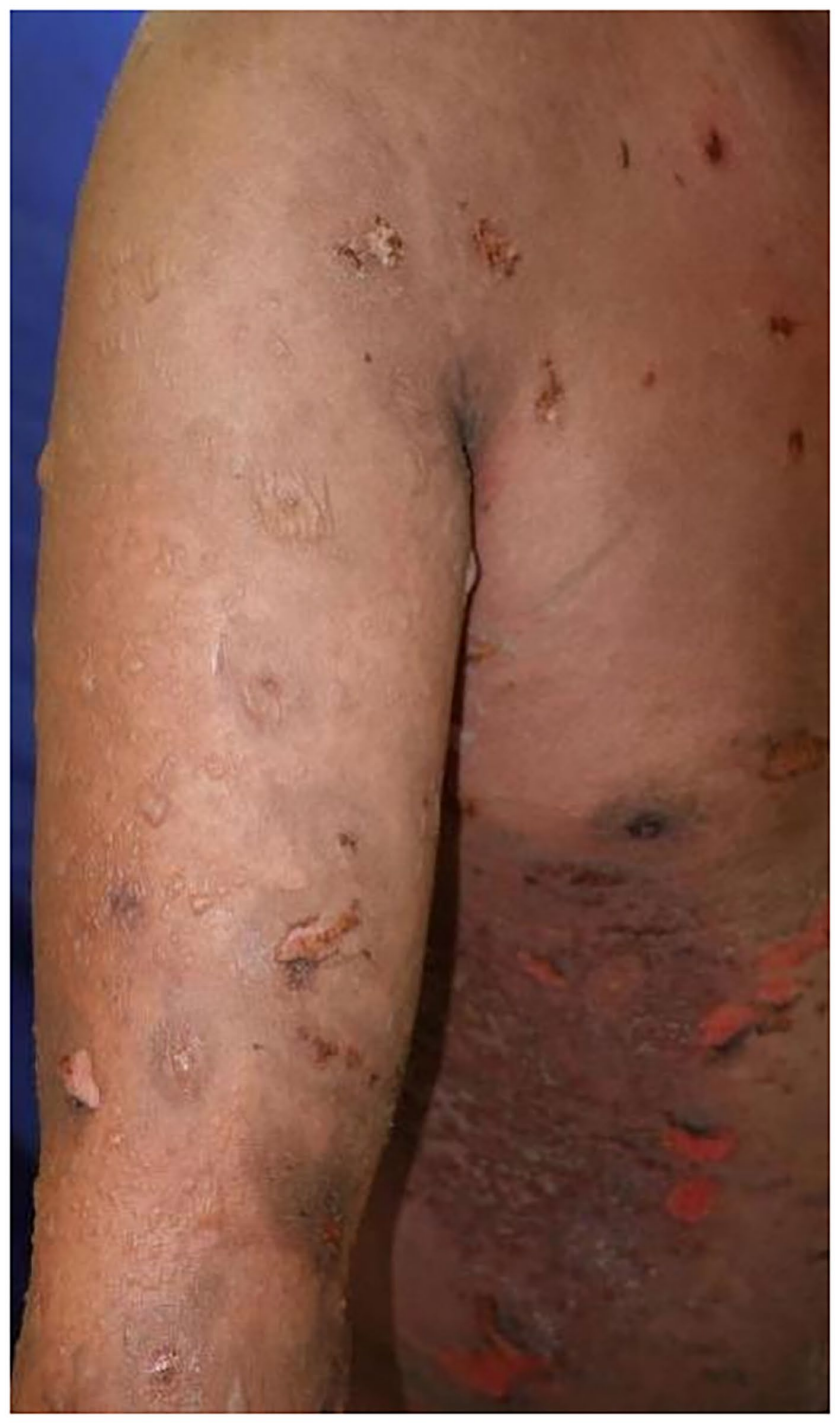
Clinical appearance of toxic epidermal necrolysis. Notice the erythematous, target-like, maculopapular rashes with blisters and bullae on the trunk and upper extremity.

**Table 2:** Changes in condition and diagnostic/treatment process.

Timeline	Symptoms and signs	Treatment and interventions	Laboratory tests and examinations
Days 1–2	Temperature: 36.5 °C Heart rate: 85 beats/min Blood pressure: 135/75 mmHg Skin and mucosa Diffuse red macules and patches on the head, face, trunk, and limbs. Large areas of epidermal detachment and erosion on the neck, back, and sacral region, with moist, reddened surfaces. Flaccid blisters and bullae on the trunk, limbs, and dorsum of the feet. Nikolsky’s sign: Positive. Significant tenderness. Total BSA with epidermal detachment: 60%. Scrotal mucosal erosion. The oral mucosa showed no obvious involvement. There is partial scrotal skin erosion with no genital mucosal involvement. The patient has normal vision in both eyes without significant discharge. Pain: At rest, experiencing mild to moderate pain (Numerical Rating Scale [NRS] 3–4)	Corticosteroids: Initial high-dose prednisolone (125 mg/day equivalent) Supportive therapy • Human immunoglobulin • potassium, calcium, and vitamin supplementation Biologic agent (only Day 2): Infliximab 500 mg IV (vital signs were closely monitored during infliximab administration for adverse reactions)	Imaging findings (chest–abdomen–pelvis CT) • Fatty liver. • Splenomegaly. • Multiple enlarged retroperitoneal and bilateral inguinal lymph nodes (some described as enlarged and rounded). Laboratory examination • White blood cells (WBCs): 12.0 × 10^9^/L • Neutrophils: 85.0% • High-sensitivity C-reactive protein (CRP): 122.40 mg/L • Alanine aminotransferase (ALT): 142 U/L • Urea: 5.48 mmol/L • Glucose: 10.1 mmol/L • Total carbon dioxide (TCO_2_): 22.7 mmol/L • Procalcitonin: 0.52 ng/mL • Erythrocyte sedimentation rate (ESR): 20 mm/h • D-dimer: 1.13 mg/L FEU • Interleukin-6 (IL-6): 20.4 pg./mL • IL-10: 11.7 pg./mL • Tumor necrosis factor-alpha (TNF-α): 20.8 pg./mL • ANA (IgG): Positive, 1:80
Day 3	Temperature: Tmax 38.2 °C Skin and Mucosa New erythema persisted on both feet and ankles. Multiple flaccid blisters throughout the body. Pain: At rest, experiencing mild to moderate pain (NRS 3–4)	Continued prior therapies Pain medication intervention: Tramadol hydrochloride sustained-release tablets 100 MG Oral Bid (8–16)	Laboratory examination • WBC: 2.83 × 10^9^/L • Neutrophils: 52.6% • ALT: 106 U/L • Urea: 5.61 mmol/L • Glu: 9.1 mmol/L • TCO_2_: 27.8 mmol/L • Procalcitonin: 0.52 ng/mL • ESR: 20 mm/h • D-dimer: 0.47 mg/L FEU
Day 5	Temperature: 36.3 °C Intake and output: 3930/1870 Skin and Mucosa Epidermal detachment area increased to 70% (Figures 1, 2). Pain: Severe pain during dressing change (NRS 8–9), patient agitated and refused to co-operate	Pain medication intervention: Starting from the next day, adjust the administration time of 100 mg tramadol hydrochloride to 8 a.m. to half an hour before the dressing change.	Laboratory examination • WBC: 6.47 × 10^9^/L • Neutrophils: 75.5% • ALT: 87 U/L • Urea: 9.10 mmol/L • Glu: 10.2 mmol/L • TCO2: 25.8 mmol/L • ESR: 60 mm/h • Local wound *Enterococcus faecalis* culture positive
Day 7	Intake and output: 3970/1400 Skin and Mucosa Epidermal detachment reached 80%. Facial crusting, shedding, and dryness were noted. Turning point: New blistering decreased, and re-epithelialization began. Pain During nighttime rest, systemic pain symptoms intensified and became unbearable (NRS 8–9).	Corticosteroids: 75 mg/day Cyclosporine: 150 mg, twice daily, orally Supportive therapy: Discontinue intravenous immunoglobulin Pain medication intervention: After consultation with the department of anesthesiology, tramadol hydrochloride was changed to MS Contin (morphine sulfate sustained-release tablets) 10 mg orally Q12 h (8–20). For sudden nocturnal pain, add Artemisia argyi, estazolam 1 mg, and tramadol hydrochloride 100 mg.	
Day 9	Temperature: Tmax 37.8 °C Intake and output: 2390/2800 Skin and Mucosa • Face: Largely re-epithelialized; healed erosions and crusts on the upper lip. •Trunk, limbs, and scrotum: Exhibit confluent erythema, erosions, and extensive epidermal detachment. Approximately 80% of the BSA is affected by superficial sloughing. •Limbs (new findings): Scattered flaccid blisters of varying sizes are present, with a partially positive Nikolsky’s sign. •Neck and distal extremities: Confluent erythematous papules are noted. Pain: During dressing changes and at night, there is still severe pain. (NRS 6–7).	Antibiotics: Cefaclor 0.75 g Q12 h po (select based on drug sensitivity results) Pain medication intervention: Pregabalin capsules 150 mg oral Qn	Laboratory examination • WBC: 7.11 × 10^9^/L • Neutrophils: 58.3% • ALT: 112 U/L • Albumin (Alb): 27 g/L • Urea: 6.73 mmol/L • Glu: 4.6 mmol/L • TCO2: 32.9 mmol/L • High-sensitivity CRP: 93.94 mg/L • Procalcitonin: < 0.072 ng/mL • Cyclosporine A (CsA_C0): 73.3 ng/mL • Bacterial culture and drug sensitivity test of skin swab from leg wound: Escherichia coli extended-spectrum beta-lactamase (ESBL) and *Pseudomonas aeruginosa*.
Day 13	Temperature: Tmax 37.8 °C Intake and output: 3640/1950 Skin and mucosa • Face: Largely re-epithelialized; the eroded/crusted area on the upper lip has healed. • Both lower limbs: Show large patches of dark erythema, with slightly elevated skin temperature and marked tenderness (highly suspected to be local symptoms caused by infection). A few fingernail-sized blisters are present; the Nikolsky’s sign is negative. • Right thigh (lateral side): Displays an eroded surface with minimal exudate. Pain: The pain condition has improved compared to before (NRS 5–6).	Corticosteroids:60 mg/day Antibiotics: Change cefaclor to cefepime 2 g Q12h IV (this is empirical medication) Supportive Therapy: Human serum albumin 30 g/day IV Pain medication intervention: Discontinue MS Contin (morphine sulfate extended-release tablets) and adjust the dosage of pregabalin capsules to Bid (8–16).	Laboratory examination • WBC: 10.32 × 10^9^/L • ALT: 70 U/L • Alb: 27 g/L • UREA: 6.74 mmol/L • Glu: 3.6 mmol/L • TCO2: 32.2 mmol/L • High-sensitivity CRP: 20.29 mg/L • D-dimer: 1.13 mg/L FEU • Fasting blood glucose (Fbg): 6.54 g/L • D-Dimer: 1.23 mg/L FEU
Day 16	Temperature: 36.5 °C Skin and mucosa: • Both lower limbs: The erythema has darkened in color compared to before, with normal skin temperature and mild tenderness upon touch. Pain: The pain condition has significantly improved (NRS ≤ 3).	Corticosteroids: 50 mg/day Supportive therapy: Discontinue human serum albumin IV	Laboratory examination • D-dimer: 0.25 mg/L FEU
Day 20 (discharge)	Skin is almost fully healed, a significant overall improvement (Figure 3).	Corticosteroids: 40 mg/day Antibiotics: Discontinue cefepime 2 g Q12h IV	Laboratory examination • WBC: 12.31 × 10^9^/L • ALT: 41 U/L • Alb: 42 g/L • Urea: 8.6 mmol/L • Glu: 5.8 mmol/L • TCO2: 32.3 mmol/L • ESR: 20 mm/h • CsA_C0: 74.6 ng/mL
Follow-up visit	General condition is good, with localized scabbing on the right thigh, BSA < 1%.		

## 3 Nursing care

### 3.1 Skin care

The skin, being the largest organ of the human body, plays protective and regulatory roles, effectively shielding tissues from harmful factors and maintaining body temperature. In TEN patients, large areas of epidermal detachment disrupt thermoregulation. Skin infections leading to sepsis are the most common cause of death in such cases (1).

#### 3.1.1 Environmental preparation

Thirty minutes before dressing changes, the responsible nurse closed the doors and windows of the patient’s room and used a heater to pre-warm the room to 25–28 °C (6), thereby enhancing patient comfort. During the procedure, the doors and windows were shut, and the curtains were drawn to protect the patient’s privacy. All necessary materials were prepared in advance to avoid prolonging the dressing change time due to missing supplies.

#### 3.1.2 Wound care

1. Upon admission, the patient had widespread bullae and blisters. For tension blisters >2 cm in diameter, aspiration was performed under strict aseptic conditions using a 20-mL syringe after routine disinfection, while preserving the blister roof whenever possible. Blisters ≤2 cm were left untreated to be absorbed spontaneously (7).
2. Wound cleansing: Debridement solution was selected based on the wound stage.
3. During the initial hospitalization period (Days 1–5), the patient was in the wound stabilization phase and received saline treatment. During the mid-treatment phase (Days 6–13), skin swab cultures successively detected *Enterococcus faecalis* (Day 5), ESBL *Escherichia coli*, and *Pseudomonas aeruginosa* (Day 9), leading to the use of a 1:8000 potassium permanganate solution. After infection control, ulcer oil was applied to facilitate debridement and alleviate patient discomfort.
4. Nurses used sterile cotton balls soaked in cleansing solution to gently wipe the erosive areas. After cleaning, mupirocin ointment was applied to sterile Vaseline gauze, which was then patch-applied to the wounds like a “map.”
5. To protect the wound and enhance the effect of mupirocin, limbs were wrapped with sterile gauze and bandages, with enough looseness to fit two fingers, allowing movement and preventing wound traction. For the chest, back, and buttocks, which are difficult to wrap, gauze clothing was used. Adhesive tape was avoided to prevent sticking to the skin.
6. Daily, a 0.01–0.02% hypochlorous acid solution was prepared for foot soaking (8) to cleanse and reduce inflammation. To minimize pain from exposed skin, each wound care session was limited to under 1 h.

#### 3.1.3 Mucosal care

The patient had significant lip mucosal involvement, making mouth opening difficult. For lip erosion and crusting, tobramycin–dexamethasone eye drops were applied via wet compress for 10 min daily, followed by tobramycin eye ointment. Both contain tobramycin for anti-infection; the eye drops also include dexamethasone for anti-inflammation (9). The patient was instructed to avoid scratching or pulling scabs and to increase mouth-rinsing frequency to maintain mucosal hygiene and moisture. By Day 8, lip erosions healed, and scabs were completely shed (Table 3).

**Figure 3:**
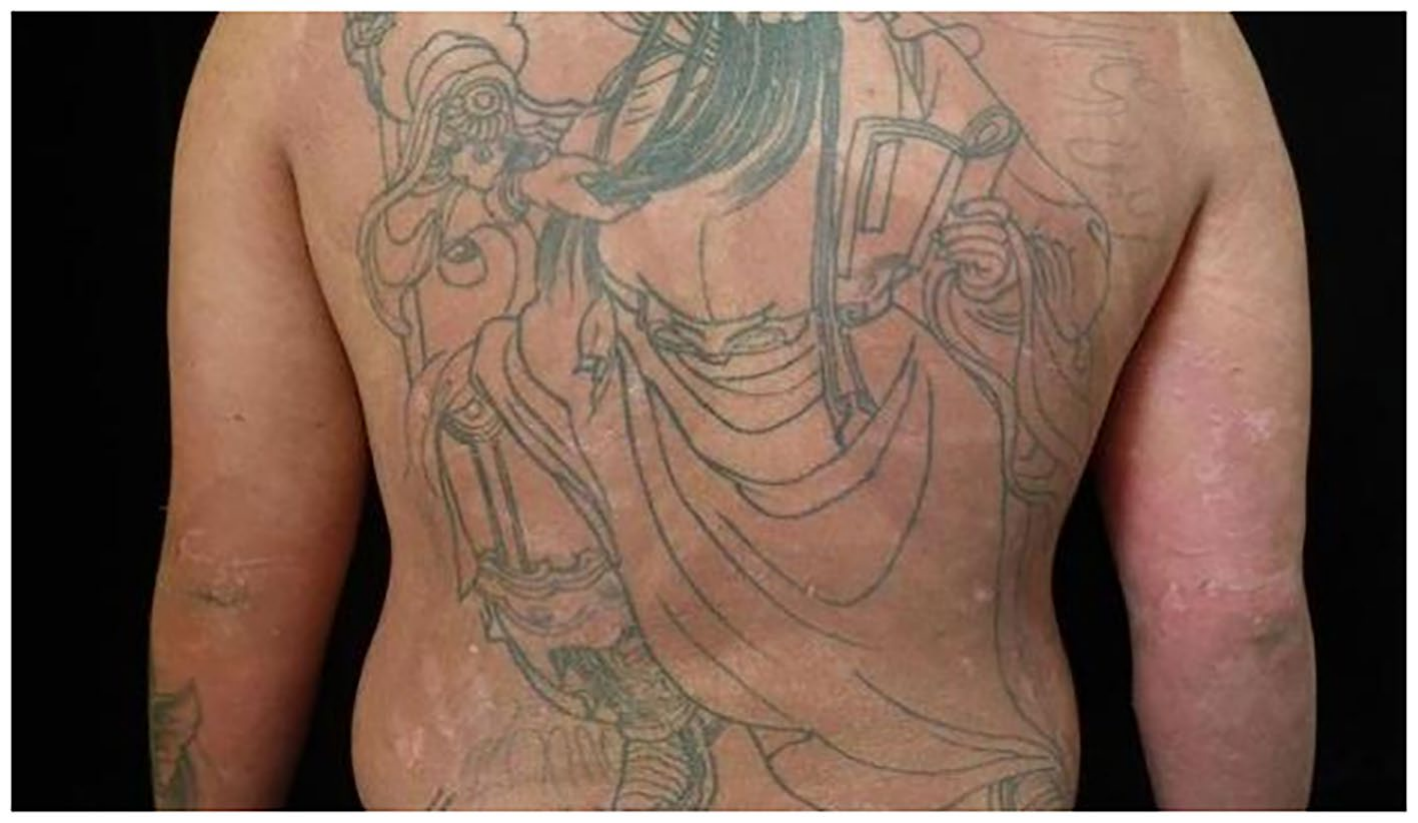
Re-epithelialization of the skin on the patient’s back.

**Table 3:** Wound staging and corresponding debridement solution selection.

Stage/period		Clinical features	Treatment	Objective
Exfoliation phase	Stable period	The epidermis has been completely avulsed or eroded, exposing a moist and erythematous wound surface without signs of infection.	Normal saline	Cleanse the skin; maintain skin hygiene.
	Infectious period	A large amount of infectious exudate, a foul odor, and a confirmed infection/high risk of infection	1:8000 potassium permanganate solution	Killing bacteria and spores; reducing or preventing infections
Re-epithelialization phase	Remission period	Exudate has decreased, no *Parazacco spilurus* subsp. spilus odor, no new epidermal exfoliation, the risk of infection is lower than the previous infection period, but the regenerated epithelium is fragile and prone to friction-induced ulceration.	Ulcer oil (a topical traditional Chinese medicine preparation, with main ingredients including rhubarb, *Conioselinum anthriscoides* ‘Chuanxiong’, Angelica dahurica, etc.)	Soften and lubricate the dressing attached to the wound; alleviate pain; promote healing.

### 3.2 Pain management

Pain was the patient’s primary issue during wound care and treatment (10), making personalized pain management essential.

#### 3.2.1 Pain assessment frequency and methods

Starting from the patient’s admission, routine pain assessment was conducted every 4 h using the Numerical Rating Scale (NRS). Additionally, dynamic evaluations of the patient’s pain response and behavioral manifestations, such as restlessness, grimacing, or protective posturing, were performed during and after each dressing-change procedure.

#### 3.2.2 Pain management goals

Our short-term goal is to maintain the patient’s pain at a mild level (NRS 1–3) to ensure adequate rest and the smooth execution of dressing changes. The long-term objective is to minimize the negative impact of pain on the patient’s emotional state and treatment compliance through multimodal analgesia.

#### 3.2.3 Analgesic interventions and adjustments

Drug interventions are detailed in Table 2.

Non-pharmacological interventions: Nursing care involved gentle dressing-change techniques (e.g., fully moistening gauze to avoid forced removal) and the provision of a “bed cradle” to reduce wound pressure and friction. Concurrently, distraction methods such as music and videos were attempted, although with limited efficacy.

Localized intensive intervention (from Day 11 onward): In response to aggravated local pain in the patient’s lower limbs, a daily regimen of 0.9% saline solution (500 mL) mixed with 5 mL of lidocaine hydrochloride was applied as a wet compress for 10 min to enhance local analgesic effects.

#### 3.2.4 Adverse effect monitoring

Starting from Day 7 after implementing the new opioid regimen, nurses intensified monitoring of the patient’s vital signs, with particular focus on opioid-related side effects such as respiratory depression. Regular liver and kidney function tests were conducted to ensure medication safety.

#### 3.2.5 Objective response evaluation

Despite the comprehensive measures taken, the patient’s pain remained at a moderate-to-severe level (NRS 6–7) during the initial intervention phase, failing to immediately achieve the target of mild pain (NRS 1–3). However, through continuous adjustments and interventions, by Day 15, the patient’s systemic and localized limb pain were significantly alleviated.

### 3.3 Supportive care

#### 3.3.1 Fluid resuscitation and electrolyte management

Due to extensive epidermal exfoliation and erosion in the patient, significant loss of water and electrolytes occurred. Therefore, the patient’s vital signs were closely monitored daily, with particular attention paid to electrolyte levels. Strict recording of intake and output was initiated when epidermal exfoliation progresses further (Day 5) to prevent severe electrolyte disorders caused by impaired skin barrier function and fluid exudation.

#### 3.3.2 Dietary care

For drug-induced TEN, discontinuation of the suspected allergen, adequate hydration, and enhanced drug excretion are key. However, this case was induced by an exogenous chemical, and the substance’s absorption and metabolism in the body were unclear. Hence, in the early stage of hospitalization, the patient was advised to drink more water to promote excretion and minimize the retention of allergens (11).

Due to lip erosion and crusting, the patient had difficulty opening his mouth and experienced pain, posing a risk of nutritional imbalance. He was encouraged to eat orally and guided to slow down chewing, increase food variety, and consume soft foods to maintain a balanced diet. TEN often involves extensive skin erosion and exudation, leading to protein and electrolyte loss. In the later stage, laboratory results revealed hypoalbuminemia (serum albumin 27 g/L). Intravenous human albumin was administered, and a high-protein diet was recommended, with increased intake of soy products and lean meat to replenish protein loss. One week later, liver and kidney functions were rechecked, and albumin levels had returned to normal.

#### 3.3.3 Complication management

Reduced activity, prolonged bed rest, and a hypercoagulable state significantly increase the risk of deep vein thrombosis (DVT) in the lower limbs (9). DVT can impair blood circulation, elevate venous pressure in the limbs, and cause swelling and pain. If the thrombus dislodges, it may result in acute pulmonary embolism, which can be life-threatening.

The patient’s D-dimer level was 1.23 mg/L FEU, and although he declined bilateral lower limb venous Doppler ultrasound, multiple high-risk factors were present: hypoalbuminemia, obesity, and prolonged immobility. With the patient’s consent, a prophylactic enoxaparin sodium injection (6,000 U, subcutaneously, once daily) was administered per physician’s orders. Through health education, the patient was informed about the causes and dangers of DVT. He was encouraged to engage in in-bed and bedside movements when physically able to avoid prolonged periods in the same position and thereby reduce the risk of thrombosis.

#### 3.3.4 Stress ulcer prevention

Due to the patient’s severe epidermal exfoliation condition and the use of glucocorticoids in the treatment regimen, these are high-risk factors for stress ulcers. To prevent digestive tract hemorrhage, we administered a proton pump inhibitor (omeprazole sodium) from the early stages of hospitalization and closely monitored the patient’s gastric fluid color, stool characteristics, and fecal occult blood tests.

## 4 Conclusion

The diagnosis of toxic epidermal necrolysis (TEN) in this case is definitive, supported by the following evidence: (1) clear history of extensive epidermal detachment (60% body surface area) following exposure to S, S-dimethyl cyanoimidodithiocarbonate (DCT); (2) characteristic histopathological findings (Although the biopsy was performed at an external hospital and the original histopathological documents were unavailable, the histopathological findings described in the external pathology report suggested interface dermatitis consistent with a drug reaction.); and (3) serological exclusion of autoimmune bullous diseases (negative relevant antibodies) with no clinical or laboratory evidence of active infection. Differential diagnoses were systematically ruled out, including infectious dermatoses (e.g., Staphylococcus scalded skin syndrome—inconsistent histopathology and absence of infection markers), autoimmune bullous diseases (minimal mucosal involvement and negative antibody panel), and paraneoplastic dermatoses (young patient age and negative initial imaging for malignancies). The collective clinical, histopathological, and serological findings conclusively support the diagnosis of TEN.

Assessment of causal relationships: the etiological attribution in this case requires cautious inference given the limitations of an incomplete medication history. Regarding pharmaceutical factors, the patient had no history of vaccination within 2 months prior to onset and self-reported no history of drug allergies. The glucocorticoids, antibiotics, and herbal medicines administered at the external hospital (with incomplete retrieval of the exact medication used) were all initiated after the appearance of skin lesions, which does not align with the typical temporal pattern of drug-induced TEN (usually 4 to 28 days from first drug exposure to onset). Moreover, the specific medications involved and their temporal correlation with disease progression remain unclear, making it difficult to attribute causality to any particular drug. In terms of chemical exposure, the patient developed skin lesions the day after contact with DCT powder, with a latency period (24 h) significantly shorter than that of typical drug-induced TEN but consistent with features of direct cytotoxicity or hypersensitivity reactions to chemicals. Despite subsequent avoidance of chemical exposure, the condition progressed to extensive systemic exfoliation (peak BSA involvement of 80%), which aligns with the “self-perpetuating” nature of TEN. Therefore, although pharmaceutical factors cannot be entirely ruled out, the strong temporal association between chemical exposure and skin lesion onset within 24 h, combined with the subsequent typical disease progression, suggests that the chemical substance (DCT powder) is more likely to be the initial triggering factor.

The prognostic assessment used the standard SCORe of Toxic Epidermal Necrolysis (SCORTEN) scoring system. Based on the clinical parameters within 24 h of admission, only “epidermal detachment area >10%” was positive (scoring 1 point), while the remaining six items (age, concurrent malignancy, heart rate, blood urea nitrogen, blood glucose, and bicarbonate) were all within normal ranges. Thus, the total score was 1, categorizing the patient into the low-risk group, corresponding to a predicted mortality rate of approximately 3.2%. The patient ultimately recovered smoothly, consistent with the low-risk score prediction, which also underscores the critical value of early and aggressive comprehensive treatment in improving patient prognosis.

In terms of treatment and nursing reflection, the successful management of this case highlights the critical role of a multidisciplinary collaborative model. The close coordination among the dermatology, anesthesiology, oral medicine, and specialized nursing teams ensured comprehensive interventions ranging from systemic therapy, pain management, infection control, to meticulous wound care. Regarding systemic treatment, the patient’s condition continued to progress despite receiving corticosteroid therapy at a local hospital, presenting with extensive skin lesions upon admission. Given the suboptimal response to initial standard treatment, we initiated a combined therapeutic regimen early during hospitalization, including a single intravenous infusion of infliximab 500 mg, continued corticosteroid administration, and IVIG. As a tumor necrosis factor-alpha (TNF-*α*) antagonist, infliximab neutralizes key inflammatory cytokines and theoretically possesses the capability to treat TEN. In this case, although epidermal detachment continued to progress during the initial phase of combination therapy, the patient’s skin lesions began to show significant improvement approximately on Day 7 following the combined treatment. While the influence of the natural disease course or concomitant glucocorticoid use cannot be entirely ruled out, the timing of improvement aligns with the pharmacokinetic characteristics of infliximab (its half-life being approximately 7–10 days), suggesting that the drug may have played a synergistic role in controlling the cytokine storm. This empirical treatment approach is consistent with the biologic therapy recommendations mentioned in expert consensus (10). In nursing practice, the team strictly adhered to evidence-based guidelines (12), implementing a series of meticulous care measures: non-invasive dressings to protect nascent epithelium, strict aseptic isolation to prevent cross-infection, systematic pain assessment and management based on NRS scores, and special attention to preventing procedure-related breakthrough pain during dressing changes. These measures provided essential support for successful wound healing. The aforementioned comprehensive interventions were crucial in ensuring the patient’s stable transition through the acute phase and achieving re-epithelialization. However, this case also revealed that even under multimodal analgesia (opioids combined with pregabalin and topical lidocaine), the patient still experienced severe breakthrough pain during procedures such as dressing changes before significant lesion healing, indicating that pain control remains challenging. This underscores the need for further exploration and optimization of holistic pain management in TEN patients, particularly in the prevention and treatment of procedure-related pain.

Implications for Occupational Health and Dermatotoxicology: Unlike common drug-induced TEN, this case was triggered by the industrial chemical intermediate DCT, highlighting the uniqueness and severity of unprotected occupational exposure (via respiratory and dermal routes) as pathogenic pathway. Its clinical manifestations align with chemically induced TEN reported in the literature (4, 5), both presenting as progressive, severe skin lesions originating from the exposure site. This case serves as a wake-up call for occupational health and safety, emphasizing the critical importance of protective measures for chemical industry workers. It also alerts clinicians and occupational disease specialists to maintain vigilance against severe cutaneous adverse reactions induced by non-pharmaceutical chemicals, enabling early identification and intervention.

Patient perspective: When reflecting on this illness experience, the patient reported that the severe pain during the initial stage of the disease and extensive epidermal exfoliation caused immense physical suffering and psychological fear. He highly commended the professionalism and patient-centered care provided by the multidisciplinary medical team, particularly the gentle handling and patient communication from the doctors and nurses, which offered crucial psychological support. At the same time, the patient expressed anxiety about returning to his original job position in the future and hoped his personal experience could serve as a warning to colleagues and management, emphasizing the full recognition and implementation of occupational protection measures.

## Data Availability

The original contributions presented in the study are included in the article/supplementary material, further inquiries can be directed to the corresponding authors.
